# P22 Fabrication methodologies for antimicrobial polypropylene surfaces with leachable and non-leachable antimicrobial agents

**DOI:** 10.1093/jacamr/dlad066.026

**Published:** 2023-06-14

**Authors:** Saleh Alkarri, Dimple Sharma, Teresa M Bergholz, Muhammad Rabnawaz

**Affiliations:** School of Packaging, Michigan State University, 448 Wilson Road, East Lansing, MI 48824-1223, USA; Food Science and Human Nutrition, Michigan State University, G.M. Trout FSHN Building, 469 Wilson Rd, Rm 234B, East Lansing, MI 48824, USA; Food Science and Human Nutrition, Michigan State University, G.M. Trout FSHN Building, 469 Wilson Rd, Rm 234B, East Lansing, MI 48824, USA; School of Packaging, Michigan State University, 448 Wilson Road, East Lansing, MI 48824-1223, USA

## Abstract

**Objectives:**

Develop a methodology for the fabrication of antimicrobial polypropylene (PP) surfaces with (i) leachable copper (II) chloride dihydrate (CuCl_2_·2H_2_O) and (ii) non-leachable magnesium hydroxide (Mg (OH)_2_) biocides.

**Methods and results:**

Two methodologies are used to develop antimicrobial PP surfaces. One method involves melt blending and subsequent injection moulding, where the biocide additives were compounded with PP and subsequently injection moulded. The other method involves the thermal embossing of antimicrobial agents on the surface of a PP substrate. The obtained biocide-bearing PP surfaces were evaluated against *Escherichia coli* K-12 MG1655 for 0, 4 and 24 h to evaluate their antimicrobial properties. The injection-moulded PP bearing 5% CuCl_2_·2H_2_O showed a 6-log reduction of *E. coli* K-12 MG1655 after 24 h, while only 1 log reduction was observed for PP bearing 5% Mg (OH)_2_. The thermally embossed PP surfaces bearing CuCl_2_·2H_2_O and Mg (OH)_2_ particles (at a concentration of 10 mg/mL) showed 3 log and 4 log reduction, respectively, against *E. coli* K-12 MG1655 after 24 h.

**Conclusions:**

The results clearly demonstrate that CuCl_2_·2H_2_O conferred antimicrobial properties to PP surfaces that were prepared by both injection moulding and thermal embossing approaches owing to the presence of leachable copper ions. In contrast, the non-leachable Mg (OH)_2_ imparted antimicrobial properties only to the surface prepared via the thermal embossing technique.

**Significance and impact:**

Plastics with leachable biocides are effective antimicrobial surfaces, but their toxicity is a major concern. This study provides a fabrication methodology for non-leachable PP-based antimicrobial surfaces that are potentially safer. In addition, this strategy can be extended to many other plastic substrates.

